# Maternal Death Surveillance and Response: A Tall Order for Effectiveness in Resource-Poor Settings

**DOI:** 10.9745/GHSP-D-17-00308

**Published:** 2017-09-27

**Authors:** Marge Koblinsky

**Affiliations:** a Global Health: Science and Practice, Associate Editor for Maternal Health, Washington, DC, USA.

## Abstract

Most countries with high maternal (and newborn) mortality have very limited resources, overstretched health workers, and relatively weak systems and governance. To make important progress in reducing mortality, therefore, they need to carefully prioritize where to invest effort and funds. Given the demanding requirements to effectively implement the maternal death surveillance and response (MDSR) approach, in many settings it makes more sense to focus effort on the known drivers of high mortality, e.g., reducing geographic, financial, and systems barriers to lifesaving maternal and newborn care.

See related article by Smith et al.

While extensive efforts to reduce maternal mortality through a maternal death surveillance and response (MDSR) investigation approach in Kenya were laudable, they fell far short in a number of ways toward having any tangible impact. In low- and middle-income countries (LMICs) with high maternal mortality, resources are better spent addressing obvious causes through interventions with proven effectiveness.

The MDSR strategy aims to improve quality of care and reduce maternal deaths by investigating individual maternal deaths and taking action to avoid remediable causes. As reported in this issue of GHSP, the MDSR (or an early variant thereof) was first introduced in Kenya in 2004.^1^ At that time, the Kenyan maternal mortality ratio (MMR) was estimated at over 700 deaths per 100,000 live births. As of 2015, a decade later, the MMR was not significantly different, at an estimated 510 per 100,000 live births.^2^ Yet there were further efforts to strengthen and implement the MDSR nationally. We explore the aims and implementation steps of the MDSR as launched by the World Health Organization (WHO) in 2013; the recent process of MDSR implementation in Kenya and its results; the long history of maternal death audits including response efforts; and the effectiveness of MDSR in other LMICs.

## WHAT IS THE MDSR?

As WHO stated in its 2013 launch of the MDSR^3^:

***The primary goal*** *of MDSR* is to eliminate preventable maternal mortality *by obtaining and strategically using information to guide public health actions and monitoring their impact.*

***The overall objectives of MDSR*** *are to provide* information that effectively guides immediate as well as longer term actions *to reduce maternal mortality; and to count* every maternal death, *permitting an assessment of the true magnitude of maternal mortality and the impact of actions to reduce it.*

In a continuous action cycle, there are 4 primary steps to the MDSR:

1. **Identification and notification on an ongoing basis:** Identification of suspected maternal deaths in facilities and communities, followed by immediate notification to the appropriate authorities.
2. **Review of maternal deaths by local maternal death review committees:** Examination of medical and nonmedical contributing factors that led to the death, assessment of avoidability and development of recommendations for preventing future deaths, and immediate implementation of pertinent recommendations.
3. **Analysis and interpretation of aggregated findings from reviews:** Reviews conducted at the district level and reported to the national level; development of priority recommendations for national action based on the aggregated data.
4. **Response and monitoring of the response:** Implementation of recommendations made by the review committee and those based on aggregated data analyses; monitoring to ensure that recommended actions are being adequately implemented. Actions can address problems at the community, facility, or multi-sectoral level.

In WHO's review of progress in MDSR implementation as of 2015,^4^ glaring gaps were found between development of a national policy for MDSR and actual implementation in-country, according to data from WHO's survey of 64 LMICs and from the WHO Maternal and Newborn, Child and Adolescent Health Policy Indicator database. According to this database, 86% of countries stated they had a national policy to notify all maternal deaths and 65% declared they had subnational maternal death review committees in place, but only 46% had a national maternal death review committee that met biannually. Although information on the Response component of MDSR was minimal in WHO's review (perhaps due to the short time since the formal launch of MDSR in 2013), challenges and barriers to MDSR implementation from 18 countries that contributed case studies were identified (Box). The WHO guideline suggests that it is best to start slowly, beginning with government facilities in urban areas. With growing know-how, strong leadership, and legal backing to ensure there is no blame leading to litigation, the MDSR may then be initiated in less advanced areas.

Glaring gaps were found between development of a national policy for MDSR and actual implementation in-country.

BOX **Country Case Study Insights on Challenges to MDSR Implementation**

- Lack of political buy-in and long-term vision
- Underreporting of suspected maternal deaths due to inefficient and incomplete systems of notification
- A blame culture in some places that inhibits health professionals and others from participating fully in the MDSR process
- Incomplete or inadequate legal frameworks
- Inadequate staff numbers, resources, and budget
- Cultural norms and practices that inhibit the operation of MDSR
- Problems of geography and infrastructure that inhibit the operation of MDSR

Source: WHO (2016).^4^

## HOW DOES THE MDSR DIFFER FROM OTHER AUDITS?

The many types of maternal audits now in use (e.g., for maternal death, maternal near-miss morbidities, specific practices such as cesarean deliveries) typically aim to improve quality of care, but the MDSR places particular emphasis on the need to respond to each maternal death with actions to prevent such deaths in the future. With an emphasis on the Surveillance component, the approach also entails notification, systematic collection, and analysis of every maternal death at all levels of the health system.^3^ Perinatal deaths, typically far more common than maternal deaths, have only recently received much-needed attention; WHO launched the perinatal death review guidelines in 2016^5^ and coined the term MPDSR—adding perinatal death reviews into the process.

MDSR places particular emphasis on the need to respond to each maternal death with actions to prevent such deaths in the future.

## WHAT IS THE MDSR IMPLEMENTATION STATUS IN KENYA?

In this issue of GHSP, Smith and colleagues of the Liverpool School of Tropical Medicine describe recent experience implementing MDSR in Kenya.^1^ Since 2007, the Liverpool School has provided financial and technical assistance to implement MDSR (and its earlier iterations) in Kenya, first at the facility level and more recently (2014) at the national level. As the authors state, ideal implementation of the MDSR requires a coordinated approach, whereby both national- and district-level stakeholders are enabled and supported to implement MDSR in a “no name, no blame” environment.

Ideal implementation of the MDSR requires a coordinated approach between national and district stakeholders in a “no name, no blame” environment.

At the national level, newly created entities (a coordinating secretariat and the MPDSR Committee) now manage and oversee the MPDSR process (note that the “P” for Perinatal is aspirational at this point but is incorporated in the official Kenyan title). The recently formed national coordinating committee set up to oversee and guide the MPDSR process includes both public and private stakeholders involved in health care, plus representatives from professional associations, regulatory bodies, multi- and bilateral partners, and civil society groups. Although how or who initiated this national committee is not made clear in the article, the process of bringing all partners together in a committee was reported as “difficult,” perhaps because running the committee remains a “gray area.”

Staff of the MPDSR Secretariat, established in 2014 within the Ministry of Health, are to retrieve facility case notes of maternal deaths and centrally collate and anonymize the notes, which are then sent to assessors for review. Assessors are professionals (including medical officers, obstetricians, pediatricians, and midwives) who investigate the deaths for causes. At the county level, it is intended that maternal death reporting and data capture efforts at the facility level be strengthened. Given the results reported in the article, however, it is evident that county-level efforts have not functioned as described in the WHO guide.^3^

The results fell well short of what is needed:

- Only 12% of the estimated maternal deaths in the nation were identified in 2014.
- Only half of these were reviewed.
- Only data from facilities were recovered (about half of all women deliver in facilities in Kenya).
- Assessments of cause were grouped rather generally, e.g., incorrect management for the diagnosis, infrequent monitoring, and prolonged observations with no action.
- Recommendations were also rather non-specific, including improving the documentation of the deaths, providing regular updates for professionals in emergency obstetric care, including resuscitation at lower levels for improved transfer, and ensuring staffing by professionals 24/7 in all facilities.
- Responsible groups were assigned to follow up; however the article does not report on what, if any, implementation of these recommendations was found to have happened.

## WHAT IS THE HISTORY OF AUDITS OF MATERNAL DEATH AND RESPONSE?

The WHO MDSR builds on long-established approaches to death notification and reviews/audits, initiated in high-income countries and later adopted for LMICs. In Sweden, the system of birth and death reporting, including maternal deaths, began in the mid-1700s. The country also reported how many of these maternal deaths could have been prevented (e.g., “avoidable maternal deaths”).^6^ In response to the findings, Sweden's Ministry of Health issued a new policy of training and strategically posting more midwives. This response has been credited with Sweden's early reduction in the MMR—dropping to the low 200s by 1900, before transfusions, antibiotics, and cesarean delivery became available.^7^ Similar efforts based on knowledge of the magnitude of maternal mortality were carried out in other northern European countries—Denmark, the Netherlands, and Norway—with similar early decreases in maternal mortality.^7^

The United Kingdom and the United States were slower to initiate registration and response efforts. In the United Kingdom, registering maternal deaths began with the investigation of puerperal fever in Aberdeen, Scotland, in the late 1800s, with confidential enquiries into maternal deaths (CEMD) formalized beginning only in the 1950s and 60s.^8^ In the United States, maternal death statistics became available from 1900 onwards, but inquiries into such deaths and any response came even later (early 1930s) when the public responded to obvious differences in maternal mortality levels with those reported from northern European countries.^7^

Around the early 2000s, many LMICs officially adopted CEMD or maternal death reviews, following on the UK method, and often with UK assistance. These efforts, as well as the system of integrated disease surveillance and response (IDSR) that has now been introduced in most African districts,^3^ have become the stepping stones for the present-day MDSR.

## WHAT IS NEEDED FOR MDSR TO BE EFFECTIVE?

Over the past 3 decades, we have made substantial gains in understanding the causes of maternal deaths^9^ and implementing policy and programmatic responses.^10^^,^^11^ So what is needed now for the MDSR to be effective in achieving its goals—improving the quality of maternal health care and reducing maternal deaths? Unfortunately, there are few examples of success in LMICs. The most oft-quoted are the national MDSR programs in Malaysia and South Africa: building on prior national efforts with CEMD, they both initiated the MDSR when their MMRs were increasing due to the rise in indirect causes of maternal deaths as well as quality of care issues. The MMRs subsequently declined through program efforts addressing the indirect causes and improved care management.^12^^,^^13^ In South Africa specifically, maternal deaths are known to have reduced from non-obstetric infections, typically AIDS-related, due to adoption of a new drug regimen. Deaths from hemorrhage related to cesarean delivery decreased in 1 state due to a 3-pronged strategy, including improving inter-facility ambulance transport, intensive district training on postpartum hemorrhage, and realignment of district hospitals that perform cesarean delivery.^13^

There are few examples of success with MDSR in low- and middle-income countries.

Other countries with MDSR implemented at large scale or nationally include Sri Lanka and Thailand, and the Indian states of Kerala and Tamil Nadu. In these sites, as well as in Malaysia and South Africa, the MMR was estimated to be low, typically well below 200, when the MDSR was implemented. This is important as lower MMRs mean that a maternal health care program is functioning and has addressed obvious common problems with some degree of effectiveness: staff are trained to improve maternal health and manage obstetric complications and are available in adequate numbers; facilities are equipped and supplied; and the skilled staff are accessible and used by women for improved health care (e.g., barriers to access, such as transport between levels of facilities, are being addressed). Programmatic gaps that still remain are likely quality of care and equity issues.

In the few places where MDSR has been implemented successfully, MMR was estimated to be low, meaning the maternal health care program had already addressed obvious common problems with some degree of effectiveness.

In numerous other countries—Ethiopia, Ghana, Indonesia, Nigeria, Rwanda, Tanzania, Uganda, and Zimbabwe, to name a few—the MDSR is now being tested at the project level.^4^ These are countries with much higher MMRs that reflect resource-poor health systems and continued barriers to access, including insufficiently available and accessible skilled providers to manage women with direct complications, let alone the increasing numbers of women with indirect complications, plus insufficient functioning facilities. To address these problems, and improve the existing availability and use of quality maternal health care for all, direct maternal health interventions (e.g., uterotonics immediately post-delivery) are urgently required, along with the means to make them more accessible. Preventive means to address unwanted or poorly timed pregnancies^14^ and to improve women's and girls' nutrition and health status remain high priorities as well.

Certainly, promoting a broad culture of assessment of maternal deaths among the professional cadres serving pregnant women and strengthening “bottom-up” approaches are worthy goals. However, in such circumstances with high mortality and where the major drivers of maternal death are well known, the required interventions do not need to be identified through the Response component of the MDSR to “discover” them; rather they can and should be directly implemented.

In places where maternal mortality is high and the major drives of death are well known, the required interventions do not need to be identified through MDSR; instead they can and should be directly implemented.

## CONCLUSION

The MDSR, as presently promoted by WHO, is a complex health systems strengthening process that takes time to complete the continuous action cycle. The Response component, now in the shadow of Surveillance, should be equally implemented and both Response and Surveillance monitored to ensure progress in achieving quality maternal health care and reduction of maternal deaths—the stated end goals of the MDSR. This requires strong leadership both nationally and at lower levels of governance; an appropriate legal framework to ensure no blame to professionals; the willingness and social norm of those involved in health care (eg., public and private; obstetricians, anesthetists, midwives, and other specialists to address indirect causes of maternal death) and health management (e.g., hospital administrators, district health officials) to participate; and a useful but minimal reporting/recording system to follow progress. When implemented well, the MDSR could increase communications among and between care providers, levels of care, and their management, as well as address systemic issues. But adequately meeting such conditions for effectiveness is a very tall order.
